# Modulation of Selective Extraction of Phenolic Compounds from *Capsicum chinense* By-Products via UAE/NADES: Effects of Hydrogen Bond Acceptor, Extraction Time and Drying Method

**DOI:** 10.3390/molecules31111931

**Published:** 2026-06-03

**Authors:** Kevin Alejandro Avilés-Betanzos, Dayra Priscila Turrén-Gutiérrez, Manuel Octavio Ramírez-Sucre, Juan Valerio Cauich-Rodríguez, Ingrid Mayanin Rodríguez-Buenfil

**Affiliations:** 1 Centro de Investigación y Asistencia en Tecnología y Diseño del Estado de Jalisco A.C., Subsede Sureste, Tablaje Catastral 31264, Km 5.5 Carretera Sierra Papacal-Chuburná Puerto, Parque Científico Tecnológico de Yucatán, Mérida 97302, Yucatán, Mexico; keaviles_al@ciatej.edu.mx (K.A.A.-B.); oramirez@ciatej.mx (M.O.R.-S.); 2 Instituto Tecnológico de Tuxtla Gutiérrez, Carretera Panamericana Km 1080, Tuxtla Gutiérrez 29050, Chiapas, Mexico; l21270786@tuxtla.tecnm.mx; 3 Centro de Investigación Científica de Yucatán, Unidad de Materiales, Calle 43 No. 130 por 32 y 34, Colonia Chuburná de Hidalgo, Mérida 97205, Yucatán, Mexico

**Keywords:** selective extraction, *Capsicum chinense*, hydrogen bond acceptor, NADES, UAE

## Abstract

Habanero pepper (*Capsicum chinense* Jacq. var. Jaguar) leaves are an underutilized by-product with potential as a source of polyphenols. This study evaluated UAE/NADES extraction as a tunable strategy to modulate selective polyphenol recovery rather than only maximizing total yield. A 2 × 3 × 2 factorial design was used to assess the hydrogen-bond acceptor (HBA) in fructose-based NADES, choline chloride (ChCl) or malic acid (MAc), ultrasound-assisted extraction (UAE) time (10, 20, and 30 min), and leaf drying method: freeze-drying (FzD) or oven-drying (OvD). Total phenolic content (TPC, Folin–Ciocalteu), antioxidant capacity (Ax, DPPH assay), and individual polyphenols by UPLC-DAD were determined. The highest TPC was obtained with ChCl from FzD leaves after 10 min of UAE (36.18 ± 0.70 mg GAE/g dry leaf). Maximum Ax was observed in OvD leaves after 30 min and was similar between HBAs (ChCl: 86.43 ± 0.65%; MAc: 86.95 ± 0.18%). UPLC-DAD confirmed compound-dependent selectivity, with catechin favored in MAc-FzD at 20 min (51.14 ± 1.07 mg/g dry leaf), chlorogenic acid in MAc-OvD at 10 min (16.05 ± 0.09 mg/g dry leaf), and quercetin + luteolin in MAc-FzD at 10 min (5.37 ± 0.05 mg/g dry leaf). This selective behavior may be associated with HBA-dependent solvent–solute affinity, polarity, hydrogen-bonding interactions, UAE-driven mass transfer, and drying-induced matrix changes. It is important to note that TPC, antioxidant capacity, and individual polyphenols showed a decoupled response, indicating that the overall spectrophotometric parameters did not necessarily reflect the polyphenol profile. Overall, the results show that UAE/NADES conditions can be directed toward target polyphenol profiles, supporting the valorization of *C. chinense* leaves as a source of tailored polyphenol extracts for future food, cosmetic, pharmaceutical, or nutraceutical applications.

## 1. Introduction

Habanero pepper (*Capsicum chinense* Jacq.) is one of the most emblematic horticultural species and has considerable economic, gastronomic, and cultural relevance, particularly in southeastern Mexico. Although most scientific and industrial attention has focused on the fruit because of its capsaicinoids, carotenoids and aromatic compounds, the aerial vegetative tissues of the plant, especially the leaves, remain largely underutilized. This is relevant because *Capsicum* species are increasingly recognized as reservoirs of diverse bioactive molecules beyond capsaicinoids, including phenolic acids and flavonoids with antioxidant, anti-inflammatory, and other health-related properties. Recent works on the genus *Capsicum* have highlighted the importance of polyphenols as major contributors to the functional value of pepper-derived materials, while also emphasizing that non-fruit tissues remain comparatively less explored than fruits and processed pepper products [1,2,3]. Likewise, broader evidence on dietary polyphenols supports their relevance as multifunctional phytochemicals associated with antioxidant effects, modulation of oxidative stress, and growing interest for food, nutraceutical, and pharmaceutical applications [4].

Within this context, habanero pepper leaves represent an attractive yet underexploited agro-industrial by-product. Leaf biomass is generated continuously during crop management and harvest cycles, particularly because scheduled pruning is carried out as part of standard cultivation practices. However, this biomass is generally discarded despite containing phenolic compounds of potential biological and technological interest. Work on *Capsicum chinense* by-products has already shown that non-edible fractions can exhibit relevant antioxidant and anti-inflammatory activities linked to their polyphenolic composition [5]. More specifically, previous studies on *C. chinense* leaf extracts have demonstrated that leaves can contain appreciable levels of total and individual polyphenols, including compounds such as catechin, rutin, quercetin, luteolin, and other polyphenols with functional potential [2,6,7].

These findings support the concept that habanero pepper leaves should not be regarded merely as agricultural waste, but rather as a plant source with value for selective phytochemical recovery and subsequent formulation into functional ingredients. Although direct evidence in *Capsicum chinense* leaves remains limited, studies in *Capsicum* leaves and plant extracts have demonstrated that these tissues are rich in bioactive flavonoids and phenolic compounds with antioxidant, anti-inflammatory, and enzyme-inhibitory properties, supporting their potential use in food and nutraceutical formulations. In addition, broader evidence from the genus *Capsicum* indicates that polyphenol-rich pepper matrices can serve as relevant raw materials for the development of functional foods and food ingredients [1,3,8].

However, the practical use of leaf polyphenols depends not only on their intrinsic composition, which is influenced by season, leaf age, and biotic and abiotic factors, but also on the extraction system employed. Conventional extraction of polyphenols often relies on hydroalcoholic solvents, methanol, acetone, or other organic solvents that may be effective analytically, yet present limitations from the perspective of sustainability, toxicity, downstream use, and regulatory acceptance. For this reason, natural deep eutectic solvents (NADES) have emerged as one of the most promising green alternatives for recovering polyphenols from plants. Recent reviews have shown that NADES combine low volatility, tunable polarity, high solubilization capacity, and improved compatibility with food and nutraceutical applications, making them especially attractive for plant polyphenol extraction [9,10]. In addition, their composition can be tailored through the selection of hydrogen bond donors and acceptors, water content, and extraction conditions, thereby modifying solvent–solute interactions and enabling greater affinity toward specific subclasses of phytochemicals rather than only maximizing total yield [11,12,13,14].

Although NADES combined with ultrasound-assisted extraction has been widely explored for the recovery of polyphenols from plant matrices, most studies have focused mainly on improving global extraction responses, such as total phenolic content, antioxidant capacity, or extraction yield. In contrast, less attention has been given to how specific NADES components, particularly the hydrogen bond acceptor, may influence the selective recovery of individual phenolic compounds and reshape the resulting phenolic profile. Therefore, in the present study, NADES + UAE is not approached as a new extraction method per se or as a conventional optimization process, but as a tunable extraction platform to modulate the selective recovery of phenolic compounds.

This tunability is particularly important because the extraction of polyphenol should not always be approached as a purely quantitative problem. In many plant matrices, the most valuable outcome is not necessarily the highest total polyphenol content, but rather the preferential recovery of target molecules with known bioactivity, stability, or formulation relevance. In this sense, NADES are especially attractive because their physicochemical properties can be modulated to alter hydrogen-bonding patterns, viscosity, polarity, and mass-transfer behavior, which directly influence the selective extraction of individual polyphenols. This concept has already been supported in other botanical systems. For example, Ianni et al. [15] demonstrated in coriander seeds that NADES composition can be systematically modulated to favor the recovery of specific polyphenols rather than only increasing a global extract yield. In that study, all solvents were based on choline chloride as the hydrogen bond acceptor, while the hydrogen bond donor was changed from citric acid to urea or glucose, which markedly altered the extractive selectivity. Under the optimized time (20 min), the ChCl:urea system coupled with UAE significantly enhanced the recovery of chlorogenic acid and for the isomer of chlorogenic acid, reaching 4.53 ± 0.47 and 0.53 ± 0.001 mg/g, respectively, whereas ChCl:glucose improved the extraction of protocatechuic, caffeic, and *p*-coumaric acids up to 131.13 ± 6.16, 269.03 ± 4.15, and 0.57 ± 0.00 mg/g, respectively. By contrast, the highest rutin concentration was obtained with the more acidic ChCl:citric acid system under maceration, reaching 0.82 ± 0.03 mg/g. These results clearly show that relatively small modifications in NADES composition can reshape the hydrogen-bonding environment, solvent polarity, and solute–solvent affinity, thereby enabling a targeted enrichment of individual polyphenols according to the desired extraction outcome. Likewise, Anmol et al. [16] showed specifically that selective extraction depends first on the chemical identity of the NADES and only then on process variables such as extraction time, molar ratio and percentage of added water. After screening 20 NADES systems and four conventional solvents, they found that changing the solvent composition markedly altered the target molecule recovered: a choline chloride-containing quaternary system, choline chloride:citric acid:urea:lactic acid (1:1:1:1), gave the highest aconitic acid yield (33.23 ± 0.21 mg/g), clearly above the methanol (14.17 ± 0.27 mg/g), whereas lactic acid:glycerol (1:1) was the most effective system for atisinium, reaching 85.73 ± 4.48 mg/g. Only after selecting the most suitable NADES, they optimize extraction time, together with solid-to-liquid ratio, temperature, and water content, confirming that time acted as a secondary modulating factor once the appropriate hydrogen-bonding environment had been established. Therefore, their study supports the idea that relatively small changes in NADES formulation can redirect solute–solvent affinity and intermolecular hydrogen-bond interactions, so that the best extraction conditions depend on the target compound rather than on a single universal solvent system.

A closely related recent study was reported by Carreón-Hidalgo et al. [17], who optimized food-grade NADES based on fructose, sorbitol, xylitol, citric acid, and water for phenolic compound extraction from *Moringa oleifera* leaves. Their work demonstrated that sweetener-based NADES can improve total phenolic recovery compared with conventional hydroethanolic extraction, while FT-IR and kinetic modeling supported the role of hydrogen-bonding interactions and mass-transfer behavior in the extraction process. However, that study focused on *Moringa oleifera* leaves, NADES formulation optimization, physicochemical characterization, and extraction kinetics, whereas the present work addresses a different agro-industrial matrix, *Capsicum chinense* leaves, and evaluates how NADES formulation, ultrasound-assisted extraction time, and drying method modulate not only total polyphenol recovery and antioxidant capacity, but also the selective extraction of individual polyphenols. Therefore, the present study complements and extends recent NADES-based leaf extraction research by shifting the emphasis from global phenolic yield and solvent characterization toward compound-specific selectivity in an underutilized *Capsicum* by-product.

In *Capsicum chinense*, this line of research is still emerging. Previous work has demonstrated that NADES-assisted extraction can enhance the recovery of polyphenol-rich extracts from habanero pepper by-products and leaves, while also preserving or improving their antioxidant-related functionality [2]. More recently, the feasibility of preserving these extracts through spray-drying microencapsulation and evaluating their digestive behavior has also been reported, indicating that leaf-derived phenolics may have practical utility beyond analytical extraction and may be incorporated into functional foods [18,19,20]. Nevertheless, an important knowledge gap remains: most studies still prioritize global responses such as total polyphenol content or antioxidant capacity, whereas fewer studies address how processing variables modulate the extraction of individual polyphenols in a selective and statistically integrated manner. This distinction is relevant because spectrophotometric responses may not mirror chromatographic composition, and conditions favoring one compound or compound family may differ from those maximizing another.

In addition to solvent composition, the structure of the plant matrix before extraction is also critical. Drying treatment can affect cellular integrity, enzyme inactivation, accessibility of vacuolar phenolics, and the susceptibility of compounds to oxidation or thermal degradation. Freeze-drying and oven-drying can therefore produce distinct extraction behaviors, especially when combined with structured solvents such as NADES. Similarly, ultrasound-assisted extraction (UAE) may further influence the extraction process through cavitation, cell disruption, and enhanced mass transfer [21,22].

Evidence from other leaf matrices further supports the relevance of drying and ultrasound time. In *Chenopodium berlandieri* leaves, Vargas-Madriz et al. [22] reported that freeze-drying preserved total phenolics, total flavonoids, and antioxidant capacity more effectively than oven drying, and that raw freeze-dried leaves contained higher amounts of individual phenols than raw oven-dried leaves; notably, catechin and kaempferol were detected in raw lyophilized leaves but not in raw oven-dried leaves, indicating that dehydration history can reshape the qualitative and quantitative phenolic profile. Likewise, in *Moringa oleifera* leaves extracted with ultrasound-assisted deep eutectic solvents, Wang et al. [23] found that extraction time had a non-linear effect: total phenolics increased up to 30 min, reaching 80.35 ± 0.90 mg GAE/g, but then decreased to 77.36 ± 0.97 mg GAE/g DL at 50 min, consistent with the idea that prolonged sonication may promote degradation or diminish extraction efficiency after an optimum is reached.

Based on this background, the novelty of the present study lies in evaluating *Capsicum chinense* leaves through a selective extraction framework in which NADES formulation, ultrasound-assisted extraction time, and drying method are considered together to modulate not only global responses, but also the individual polyphenol profile.

Therefore, the aim of this study was to evaluate how the hydrogen bond acceptor used in NADES formulation, extraction time under ultrasound-assisted extraction, and leaf drying method (type) modulate the recovery of total polyphenols, antioxidant capacity, and individual polyphenols from habanero pepper (*Capsicum chinense* Jacq.) leaves.

## 2. Results

### 2.1. Effect of Hydrogen Bond Acceptor, Extraction Time, and Drying Type on the Modulation of Total Polyphenol Content and Antioxidant Capacity

**Figure 1:**
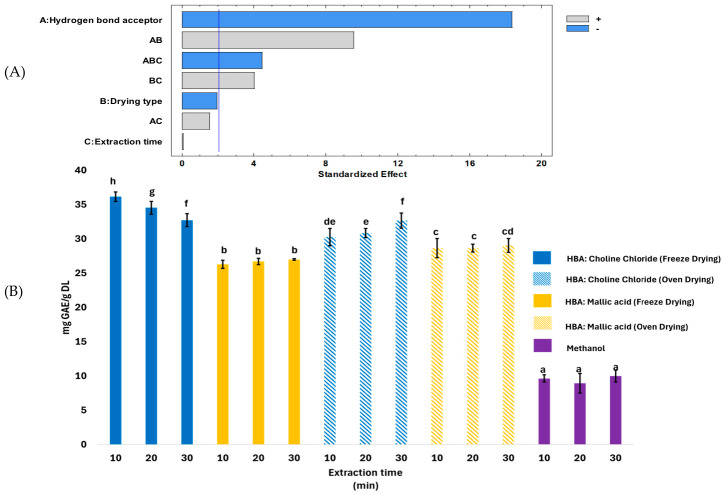
(**A**) Pareto chart showing the effect of hydrogen bond acceptor, drying method, and extraction time on total polyphenol content. (**B**) Total polyphenol content in habanero pepper leaf extracts obtained at different extraction times. Different letters indicate statistically significant differences according to the LSD test, *p* < 0.05; n = 3; Vertical blue line at Pareto chart represents the statistical significance threshold for the standardized effects.

Figure 1A shows that total phenolic content was significantly affected by the three-way interaction among HBA × ExT × DMe *(p* = 0.0001). In addition, the two-way interactions HBA × ExT *(p* < 0.0001) and ExT × DMe *(p* = 0.0004) also influenced the concentration of individual polyphenols in habanero pepper leaf NADES-based extracts. Although HBA was evaluated as an individual factor, it showed the most pronounced effect on TPC *(p* < 0.0001).

Choline chloride (ChCl) as the HBA promoted higher TPC values than malic acid under both drying conditions, reaching 36.18 ± 0.70 mg GAE/g DL in freeze-dried samples and 29.08 ± 1.20 mg GAE/g DL in oven-dried (OvD) samples. In agreement with this statistical effect, Figure 1B shows the total polyphenol content (TPC) obtained from the experimental design (Table 2).

Overall, freeze-dried (FzD) habanero pepper leaf samples extracted with ChCl exhibited the highest TPC values, although these showed a decreasing trend as extraction time increased, whereas oven-dried (OvD) samples obtained with the same HBA showed the second-highest TPC values, with a linear increase as extraction time increased.

In this case, habanero pepper leaf extracts prepared with ChCl:fructose (Fru) showed the highest concentration *(p* < 0.05) when sonication was applied for only 10 min (36.18 ± 0.70 mg GAE/g DL), with a decreasing trend after 30 min of sonication (32.76 ± 0.93 mg GAE/g DL) (Table 2, Figure 1B).

Extracts obtained with malic acid as HBA under freeze-drying conditions showed the lowest concentrations within the experimental design. Methanolic extract used as a control presented a low TPC (8.97 ± 1.44 mg GAE/g DL) compared with all extracts obtained using NADES, regardless of drying method and extraction time (Figure 1B).

Finally, the drying type (DTy) showed an opposite behavior depending on the type of HBA. In the case of ChCl, freeze-dried samples exhibited a higher concentration of polyphenols than oven-dried samples, whereas for malic acid, oven-dried samples showed a higher polyphenol concentration than freeze-dried samples. Therefore, the best conditions to be considered for achieving a high extraction of total polyphenols were the use of choline chloride as the hydrogen bond acceptor, freeze-drying, and an extraction time of 10 min (Figure 1B).

**Figure 2:**
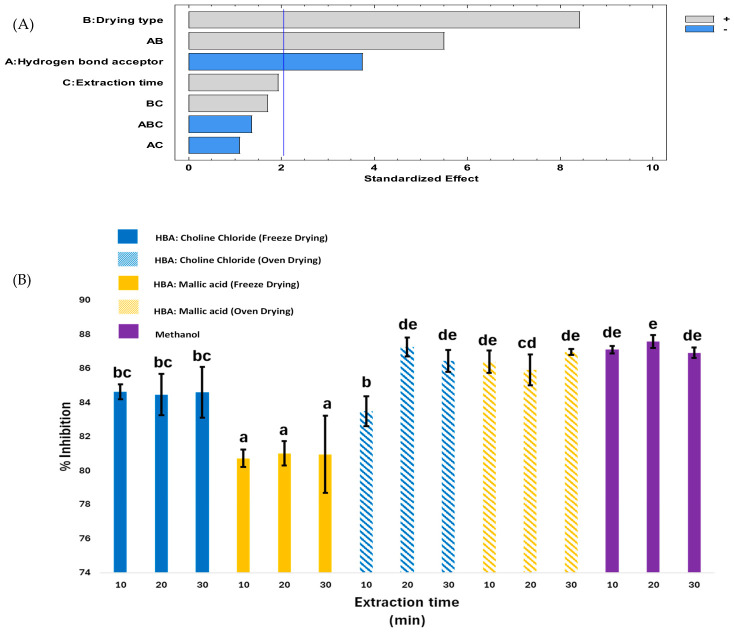
(**A**) Pareto chart showing the effect of hydrogen bond acceptor, drying method, and extraction time on antioxidant capacity. (**B**) Antioxidant capacity in habanero pepper leaf extracts at different extraction times. Different letters indicate statistically significant differences according to the LSD test (*p* < 0.05; n = 3); Vertical blue line at Pareto chart represents the statistical significance threshold for the standardized effects.

Regarding antioxidant capacity (Ax, % Inhibition DPPH), the greatest effect on this response variable (Figure 2A) was attributed to the drying type factor (*p* = 0.0001). As with TPC, the HBA also acted as a main factor *(p* = 0.001). Unlike TPC, however, Ax was affected only by the two-factor interaction between DTy and HBA *(p* = 0.0001).

The antioxidant capacity (Ax, % Inhibition DPPH) of the extracts obtained at different extraction times, using different hydrogen bond acceptors (HBA) and drying methods, is shown in Figure 2B. Overall, oven-dried samples exhibited higher Ax values than freeze-dried samples, particularly when MAc was used as HBA. In freeze-dried samples, ChCl extracts showed relatively stable Ax values across extraction times, with no marked changes from 10 to 30 min. In contrast, freeze-dried samples obtained with MAc showed lower Ax values, which remained practically unchanged as the extraction time increased, suggesting that extending the extraction time did not improve antioxidant capacity under these conditions.

For oven-dried samples, ChCl extracts showed a slight increase in Ax when the extraction time was extended from 10 to 20 min, followed by a small decrease at 30 min. Conversely, MAc extracts maintained high Ax values throughout the evaluated extraction times, with the highest values observed at 10 and 30 min. These results indicate that the effect of extraction time depended on both the HBA and the drying method; however, extending the extraction time did not consistently enhance Ax. Therefore, drying method and HBA appear to be more relevant factors than extraction time for achieving extracts with higher antioxidant capacity (Figure 2B).

On average, considering both HBA and ExT, oven-dried samples showed an Ax of 85.90 ± 1.43% inhibition, whereas freeze-dried samples exhibited an average Ax of 82.89 ± 2.07% inhibition. Finally, the methanolic extracts showed an Ax of 87.19 ± 0.28% inhibition (values calculated from the data presented in (Table 2). These results suggest that oven drying favored the recovery of extracts with higher antioxidant capacity, particularly when combined with MAc, although the Ax values were comparable to those obtained with methanolic extracts.

### 2.2. Individual Polyphenolic Profile by Hydrogen Bond Acceptor, Extraction Time, and Drying Method from Habanero Pepper Leaves

**Figure 3:**
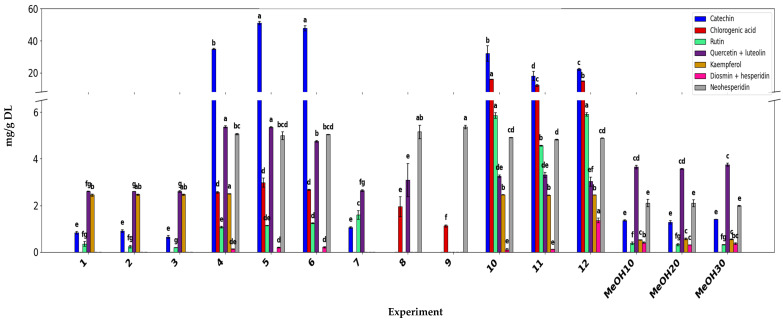
Effect of hydrogen-bond acceptor, drying method, and extraction time on the individual polyphenolic composition of habanero pepper leaves. Exp. 1–12 correspond to the factorial combinations of hydrogen-bond acceptor, drying method, and ultrasound-assisted extraction time described in Table 2. MeOH10, MeOH20, and MeOH30 correspond to methanolic control extracts obtained after 10, 20, and 30 min of ultrasound-assisted extraction, respectively. Different letters indicate statistically significant differences according to the LSD test (*p* < 0.05; n = 3).

Figure 3 shows the individual polyphenolic profile obtained from the experimental design (Table A1). Overall, the extraction conditions markedly modulated the concentration of each compound, confirming that the evaluated factors did not affect all polyphenols in the same way. In general, catechin, chlorogenic acid, rutin, quercetin + luteolin, and neohesperidin were the predominant compounds in the NADES extracts, whereas diosmin + hesperidin was detected at comparatively low concentrations. In contrast, the methanolic control extracts showed low concentrations of catechin, rutin, kaempferol, diosmin + hesperidin and neohesperidin compared with NADES extracts from the experimental design. Neohesperidin (2.11 ± 0.11 mg/g DL) and quercetin + luteolin (3.65 ± 0.05 mg/g DL) were the predominant polyphenols, whereas chlorogenic acid was not detected in these control samples.

**Figure A1:**
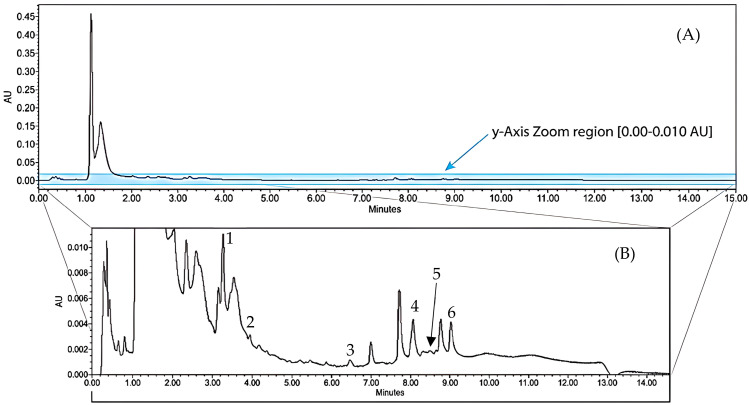
(**A**) Chromatogram of the polyphenolic profile (identified and quantified) of freeze-dried habanero pepper leaves extracted with malic acid for 20 min (Experiment 5); (**B**) Y-axis chromatogram zoom region from 0 to 0.01 AU. 1 = Catechin (3.527 min); 2 = Chlorogenic acid (3.936 min); 3 = Rutin (6.470 min); 4 = Diosmin + hesperidin (8.059 min); 5 = Neohesperidina (8.467 min); 6 = Quercetin + luteolin (9.018 min).

More specifically, catechin showed the greatest variation among experiments (Table A2). The highest catechin concentrations were observed in freeze-dried samples extracted with malic acid (MAc) as the hydrogen bond acceptor, particularly at 20 min (Exp. 5; 51.14 ± 1.07 mg/g DL; Figure A1) and 30 min (Exp. 6; 47.88 ± 1.74 mg/g DL). These values were significantly higher *(p* < 0.05) than those obtained in freeze-dried samples extracted with MAc for 10 min (Exp. 4) and in oven-dried samples extracted with MAc for 10 min (Exp. 10) and 20 min (Exp. 11). In contrast, very low or undetectable catechin concentrations were recorded in freeze-dried samples extracted with choline chloride (ChCl) for 10–30 min (Exp. 1–3) and in oven-dried samples extracted with ChCl for 10–30 min (Exp. 7–9). MeOH controls, in turn, only reached values between 1.28 and 1.41 mg/g DL.

Likewise, quercetin + luteolin was favored in freeze-dried samples extracted with MAc for 10 min (Exp. 4; 5.37 ± 0.04 mg/g DL) and 20 min (Exp. 5; 5.36 ± 0.02 mg/g DL), followed by freeze-dried samples extracted with MAc for 30 min (Exp. 6; 4.75 ± 0.03 mg/g DL). These values were significantly higher than those observed in freeze-dried samples extracted with ChCl for 10–30 min (Exp. 1–3) and in oven-dried samples extracted with ChCl for 10 min (Exp. 7) *(p* < 0.05).

**Figure A2:**
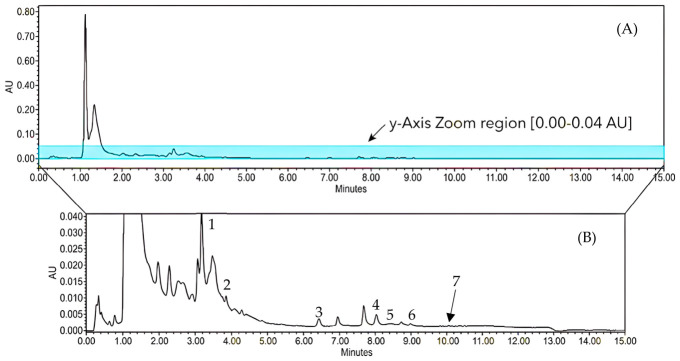
(**A**) Chromatogram of the polyphenolic profile (identified and quantified) of oven-dried habanero pepper leaves extracted with malic acid for 20 min (Experiment 11). (**B**) Y-axis chromatogram zoom region from 0 to 0.04 AU. 1 = Catechin (3.527 min); 2 = Chlorogenic acid (3.936 min); 3 = Rutin (6.470 min); 4 = Diosmin + hesperidin (8.059 min); 5 = Neohesperidina (8.467 min); 6 = Quercetin + luteolin (9.018 min); 7 = Kaempferol (10.089 min).

Chlorogenic acid and rutin showed a different response pattern (Figure 3). Chlorogenic acid was not detected in freeze-dried samples extracted with choline chloride (ChCl) for 10–30 min (Exp. 1–3), in oven-dried samples extracted with ChCl for 10 min (Exp. 7), or in any of the methanolic controls (Figure 3). However, its concentration increased markedly in oven-dried samples, particularly when MAc was used as the hydrogen bond acceptor. The highest concentration was observed in oven-dried samples extracted with MAc for 10 min (Exp. 10; 16.05 ± 0.13 mg/g DL; Figure A2), followed by oven-dried samples extracted with MAc for 30 min (Exp. 12; 14.94 ± 0.07 mg/g DL) and 20 min (Exp. 11; 12.18 ± 0.62 mg/g DL). These values were significantly higher than those obtained in freeze-dried samples extracted with MAc for 10–30 min (Exp. 4–6), freeze-dried samples extracted with ChCl for 10–30 min (Exp. 1–3), and oven-dried samples extracted with ChCl for 10–30 min (Exp. 7–9) *(p* < 0.05). A similar trend was observed for rutin, whose highest concentrations were recorded in oven-dried samples extracted with MAc for 30 min (Exp. 12; 5.92 ± 0.08 mg/g DL) and 10 min (Exp. 10; 5.86 ± 0.12 mg/g DL). These values were significantly higher than those detected in the freeze-dried treatments and in the MeOH controls *(p* < 0.05), confirming that oven drying strongly favored rutin recovery under selected MAc-based NADES conditions.

Regarding neohesperidin, this compound was absent in freeze-dried samples extracted with ChCl for 10–30 min (Exp. 1–3) and in oven-dried samples extracted with ChCl for 10 min (Exp. 7), but it became one of the major constituents in most of the remaining NADES extracts. The highest concentration was found in oven-dried samples extracted with ChCl for 30 min (Exp. 9; 5.37 ± 0.07 mg/g DL), followed by oven-dried samples extracted with ChCl for 20 min (Exp. 8; 5.16 ± 0.29 mg/g DL). In addition, consistently high concentrations were also observed in freeze-dried samples extracted with MAc for 10–30 min (Exp. 4–6) and in oven-dried samples extracted with MAc for 10–30 min (Exp. 10–12), ranging from 4.82 to 5.06 mg/g DL (Figure 3).

In comparison, the methanolic controls contained notably lower neohesperidin concentrations (1.99–2.11 mg/g DL). Kaempferol showed a more restricted distribution, being detected mainly in freeze-dried samples extracted with ChCl for 10–30 min (Exp. 1–3), freeze-dried samples extracted with MAc for 10 min (Exp. 4), and oven-dried samples extracted with MAc for 10–30 min (Exp. 10–12), with concentrations close to 2.45–2.51 mg/g DL, whereas it was not detected in freeze-dried samples extracted with MAc for 20–30 min (Exp. 5–6) or in oven-dried samples extracted with ChCl for 10–30 min (Exp. 7–9). The methanolic controls showed much lower kaempferol concentrations (0.53–0.57 mg/g DL). Finally, diosmin + hesperidin was the least abundant compound overall and was absent in several experimental runs; however, its highest concentration was found in oven-dried samples extracted with MAc for 30 min (Exp. 12; 1.36 ± 0.10 mg/g DL), which was significantly higher than the remaining experimental conditions and the methanolic controls *(p* < 0.05, Table 2).

These results indicate that the extraction conditions modulated the polyphenols in a selective manner. Freeze-dried samples were particularly favorable for catechin and quercetin + luteolin enrichment, whereas oven-dried samples, especially those obtained with malic acid, favored the accumulation of chlorogenic acid, rutin, and neohesperidin. Therefore, the best extraction conditions depended on the target compound rather than on a single global response.

**Figure 4:**
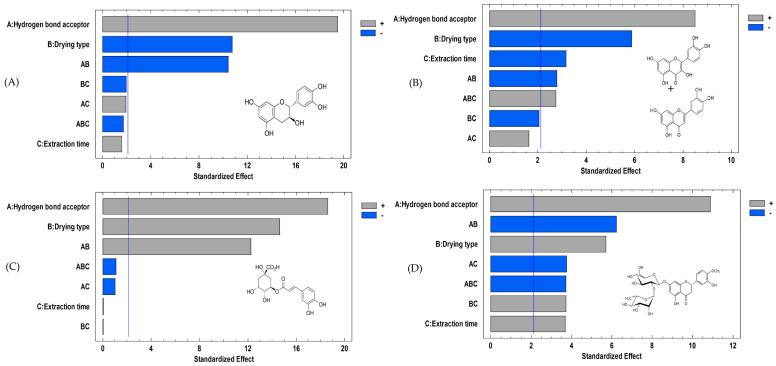
Pareto chart of (**A**) Catechin, (**B**) Quercetin + luteolin, (**C**) Chlorogenic acid and (**D**) Neohesperidin. Vertical blue line at Pareto chart represents the statistical significance threshold for the standardized effects.

The standardized Pareto charts shown in Figure 4 indicate that, in all cases, the hydrogen bond acceptor (factor A), drying type (factor B), and their interaction (AB) had significant effects on the extraction of the major compounds. This common pattern confirms that solvent system and drying method were the main factors governing the selective recovery of polyphenols from habanero pepper leaves. However, differences were observed for the remaining factors and interactions depending on the compound evaluated. For catechin (Figure 4A), only factors A and B and the AB interaction exceeded the significance threshold, whereas extraction time and the other interactions did not show significant effects. A similar trend was observed for chlorogenic acid (Figure 4C), indicating that its extraction was mainly controlled by the hydrogen bond acceptor, drying type, and the interaction between both factors. In contrast, quercetin + luteolin (Figure 4B) showed a more complex response, since, in addition to A, B, and AB, extraction time (C) and the three-way interaction (ABC) also had significant effects. Neohesperidin (Figure 4D) exhibited the most complex behavior, as all three main factors and all interaction terms significantly affected its extraction. Among them, factor A showed the largest standardized effect, followed by AB and factor B.

Although rutin was also one of the major compounds detected in the extracts, its statistical behavior was less complex than that of the other predominant polyphenols. According to Table 1, rutin concentration was significantly affected only by the hydrogen bond acceptor (factor A) and drying type (factor B), whereas extraction time (factor C) did not exert a significant main effect. Likewise, rutin did not show significant responses to the AC, BC, or ABC interactions, indicating that its recovery depended primarily on solvent identity and matrix pre-treatment rather than on sonication time or on other combinations of factors.

Overall, the results shown in Figure 4 demonstrate that the three experimental factors modulated the polyphenolic composition in a compound-dependent manner. Thus, the conditions that favored the extraction of catechin were not necessarily the same as those maximizing chlorogenic acid, quercetin + luteolin, or neohesperidin. This selective behavior may explain the differences observed in Figure 3 and highlights the importance of considering individual polyphenols, rather than only global responses, when defining the conditions associated with the best extraction.

The statistical significance of the main factors and their interactions on total polyphenol content (TPC), antioxidant capacity (Ax), and the quantified individual polyphenols is summarized in Table 1. Overall, the hydrogen bond acceptor (factor A) was the only factor that significantly affected all response variables *(p* < 0.05), confirming its central role in modulating both the global responses and the individual polyphenol profile. Drying type (factor B) also showed a significant effect on most variables, including Ax, catechin, chlorogenic acid, rutin, quercetin + luteolin, kaempferol, diosmin + hesperidin, and neohesperidin *(p* < 0.05), although its effect on TPC was not significant *(p* = 0.0630). In contrast, extraction time (factor C) had a more selective influence, significantly affecting quercetin + luteolin, kaempferol, diosmin + hesperidin, and neohesperidin *(p* < 0.05), but not TPC, Ax, catechin, chlorogenic acid, or rutin.

Regarding factor interactions (Table 1), the two-factor interaction between hydrogen bond acceptor and drying type (AB) significantly affected all response variables *(p* < 0.05), indicating that the effect of one factor depended strongly on the level of the other. This interaction was particularly consistent across both the spectrophotometric responses and the chromatographically quantified compounds. By contrast, the interaction between hydrogen bond acceptor and extraction time (AC) showed a more limited effect, being significant only for kaempferol, diosmin + hesperidin, and neohesperidin *(p* < 0.05). Similarly, the interaction between drying type and extraction time (BC) did not significantly influence TPC, Ax, catechin, chlorogenic acid, rutin, or quercetin + luteolin *(p* > 0.05), but it significantly affected kaempferol, diosmin + hesperidin, and neohesperidin *(p* < 0.05).

Finally, the three-way interaction (ABC) was significant for TPC, rutin, quercetin + luteolin, kaempferol, diosmin + hesperidin, and neohesperidin *(p* < 0.05), whereas no significant three-factor effect was detected for Ax, catechin, or chlorogenic acid *(p* > 0.05). These results indicate that the modulation of the polyphenol profile was compound-dependent and statistically more complex than the modulation of TPC and Ax (Table 1).

Kaempferol, diosmin + hesperidin, and neohesperidin were the most sensitive compounds, as all three main factors and all interaction terms significantly influenced their extraction behavior. Overall, these findings confirm that the conditions leading to the best extraction depend on the target response variable and that the selective recovery of individual polyphenols cannot be inferred solely from global responses such as TPC or Ax.

### 2.3. Heatmap and Hierarchical Clustering Analysis of Treatments Based on TPC, Ax, and Polyphenolic Composition

**Figure 5:**
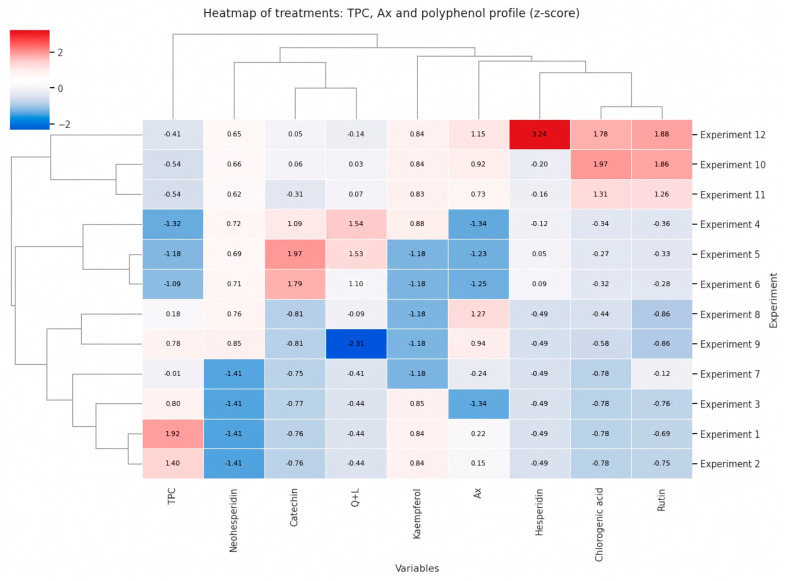
Hierarchical clustering heatmap of the experimental treatments based on z-score standardized values of total polyphenol content (TPC), antioxidant capacity (Ax), and the individual polyphenols identified in habanero pepper leaf extracts.

To further explore the relationship among the response variables and the experimental treatments, a hierarchical clustering heatmap based on z-score standardized data was constructed (Figure 5). This analysis revealed a clear separation of the treatments into groups with distinct polyphenol signatures, confirming that the extraction conditions modulated not only the concentration of individual compounds but also the overall response pattern integrating TPC, Ax, and the polyphenolic profile. In general, positive z-scores were associated with the enrichment of specific variables within a treatment, whereas negative z-scores indicated relative depletion.

At the treatment level, the dendrogram separated four main clusters. The first cluster grouped Exp. 10, Exp. 11, and Exp. 12, which corresponded to oven-dried samples and were characterized by high relative values of chlorogenic acid, rutin, and antioxidant capacity, together with the highest enrichment of diosmin + hesperidin, particularly in Exp. 12. This clustering pattern agrees with the individual results shown in Figure 3, where these treatments exhibited the highest concentrations of chlorogenic acid and rutin, confirming that oven drying, especially under malic acid-based conditions, promoted a differentiated polyphenol composition. A second cluster comprised Exp. 4, Exp. 5, and Exp. 6, corresponding to freeze-dried samples extracted for 20–30 min. These treatments were mainly associated with high catechin and quercetin + luteolin values, and particularly Exp. 5 and Exp. 6 showed the strongest positive z-scores for catechin. This pattern supports the selective enrichment of flavonoids under freeze-drying conditions already observed in the chromatographic analysis.

A third cluster included Exp. 8 and Exp. 9, which were mainly associated with high antioxidant capacity and neohesperidin, but low catechin, kaempferol, chlorogenic acid, and rutin. This indicates that these treatments generated extracts with a more restricted but still distinctive polyphenol pattern, in which antioxidant performance was more closely linked to specific compounds than to a broad polyphenol enrichment. In contrast, Exp. 1, Exp. 2, and Exp. 3 formed a separate cluster characterized by relatively high TPC and kaempferol, but low chlorogenic acid, rutin, neohesperidin, and diosmin + hesperidin. Exp. 7 appeared close to this branch, although it showed a more depleted profile overall, particularly for neohesperidin and the other major compounds, suggesting that this condition was less favorable for selective polyphenol recovery.

At the variable level, the dendrogram also revealed meaningful associations among the response variables (Figure 5). Chlorogenic acid and rutin clustered closely together, indicating that both compounds followed a similar response pattern across treatments, particularly in oven-dried samples. Catechin and quercetin + luteolin are also grouped within the same branch, which is consistent with their co-enrichment in Exp. 4–6. By contrast, TPC and Ax did not cluster directly with the same individual compounds, reinforcing that global spectrophotometric responses do not necessarily reflect the behavior of the dominant polyphenols detected by UPLC. Overall, the heatmap and hierarchical clustering analysis confirmed that the evaluated extraction conditions generated differentiated polyphenol fingerprints, and that the optimal treatment depended on whether the objective was to maximize global responses such as TPC and Ax or to selectively enrich specific polyphenolic compounds.

Overall, these results show a clear decoupling between TPC, antioxidant capacity, and individual polyphenols, indicating that conditions favoring global polyphenol recovery or antioxidant capacity did not necessarily promote the highest recovery of specific polyphenols. Therefore, chromatographic profiling was essential to identify selective extraction behavior beyond the information provided by global spectrophotometric responses.

## 3. Discussion

The present results confirm that habanero pepper leaves are a phenolic-rich by-product whose extraction profile can be selectively redirected by solvent chemistry and process conditions. Rather than producing only a general increase or decrease in global responses, the NADES systems evaluated here modulated the polyphenol profile in a compound-dependent manner. This agrees with previous work on *Capsicum chinense* leaves, where extraction medium polarity influenced polyphenol recovery and antioxidant activity; under UAE with 50% MeOH, the highest TPC reached 24.39 ± 2.41 mg GAE/g dry leaves (DL), and the recovered profile included N-caffeoyl putrescine and apigenin-, luteolin-, and diosmetin-derived compounds [6]. In the present study, the best ChCl-based treatment reached 36.18 ± 0.70 mg GAE/g DL, while the corresponding oven-dried extract reached 29.08 ± 1.20 mg GAE/g DL, approximately 48% and 19% higher, respectively, than the best value reported with 50% MeOH. In addition to improving TPC, NADES enabled selective enrichment of individual compounds, as shown by the high concentrations of catechin (51.14 ± 1.07 mg/g), chlorogenic acid (16.05 ± 0.09 mg/g), and quercetin + luteolin (5.37 ± 0.05 mg/g) obtained under specific combinations of hydrogen-bond acceptor, drying method, and extraction time. This behavior is consistent with previous NADES-based extraction from *C. chinense* leaves, where changes in choline chloride:glucose molar ratio and added water modified TPC, DPPH inhibition, and individual polyphenol recovery, including vanillin, ferulic acid, catechin, chlorogenic acid, quercetin + luteolin, kaempferol, diosmin + hesperidin, and neohesperidin [7]. Together, these findings support that structured solvents can modulate not only the magnitude of TPC but also the relative abundance of target polyphenol in habanero pepper leaves. In the present study, however, the most notable outcome was not only the superiority of NADES over the methanolic control for TPC, but also the marked decoupling between TPC, Ax, and individual polyphenols, particularly catechin, chlorogenic acid, quercetin + luteolin, rutin, and neohesperidin.

This selective behavior is also consistent with reports in other leaf matrices. In *Moringa oleifera* leaves, Wu et al. [24] showed that DES-UAE outperformed conventional extraction under optimized conditions of 37% water in the DES, 144 W, and 40 °C; HPLC analysis identified 14 individual polyphenols, with orientin and vicenin-2 as the major metabolites at 23.6 and 17.6 mg/g, respectively. Although these compounds differ from those detected in the present work, their concentrations were lower than the highest catechin value obtained here (51.14 ± 1.07 mg/g DL) and comparable to the best chlorogenic acid concentration (16.05 ± 0.13 mg/g DL), supporting the ability of DES/NADES systems to selectively concentrate specific polyphenol families depending on the plant matrix. Similarly, Wang et al. [23] reported that seven DES systems extracted more TPC from *M. oleifera* leaves than 70% ethanol, with the organic solvent yielding 36.27 ± 1.58 mg GAE/g DL and the best DES, ChCl:citric acid, reaching 86.92 ± 1.34 mg GAE/g DL [23,24].

A more mechanistically relevant comparison for the present work is the effect of NADES chemical identity, particularly changes in the hydrogen-bond acceptor or dominant eutectic-forming component, on extraction selectivity. In blueberry leaves, Santos-Martín et al. [25] showed that different NADES systems strongly modified both extraction efficiency and polyphenol selectivity under UAE. The optimized lactic acid:sodium acetate:water (3:1:2) and ChCl:oxalic acid (1:1) systems reached 142 and 195.5 mg GAE/g DL, respectively, compared with 86.9 mg GAE/g DL using 80% methanol, and showed different selectivity toward hydroxycinnamic acids, flavonol derivatives, and anthocyanins. More directly, Fanali et al. [26] screened DESs based on choline chloride or betaine as HBAs for chlorogenic acid extraction from spent coffee grounds, while García-Roldán et al. [27] showed that ChCl:1,2-propanediol achieved higher overall polyphenol extraction than betaine:triethylene glycol, corresponding to 14 and 11 mg/g DW, respectively. In the same study, the choline-based system also improved the recovery of 3-O-caffeoylquinic acid and caffeic acid, reaching 0.13 and 0.6 mg/g, respectively, compared with 0.11 and 0.06 mg/g for the betaine-based NADES. Taken together, these studies support that NADES identity, including the hydrogen-bond acceptor environment, can reshape solvent–solute interactions and modulate both the magnitude and selectivity of polyphenol extraction.

From a molecular standpoint, the marked role of the hydrogen-bond acceptor-related solvent system is chemically plausible and can be interpreted by integrating the present results with recent experimental, mechanistic, and computational studies on DES/NADES–polyphenol interactions. Carreón-Hidalgo et al. [17] showed that modifying the sweetener component in citric acid/water NADES changed viscosity, diffusivity, and equilibrium extraction capacity, indicating that solvent composition affects both mass-transfer behavior and solubilization capacity. However, beyond these macroscopic properties, the extraction of phenolics by DES/NADES may be influenced by specific intermolecular interactions. Cao et al. [28] demonstrated that the extraction mechanism of flavonoids from plant matrices involves strong non-covalent interactions between the DES network and flavonoid functional groups, particularly hydrogen bonding with phenolic hydroxyl and carbonyl sites. Similarly, Lazović et al. [29], using COSMO-RS, showed that NADES can be rationalized as supramolecular HBD:HBA:water systems whose extraction performance depends on the interaction pattern between the eutectic network and polyphenols. In this context, choline chloride-based DES/NADES are especially relevant because the chloride anion has been reported to act as a strong hydrogen-bond acceptor and may serve as an interaction site for hydrogen-bond donation from polyphenolic hydroxyl groups, water, and the second solvent component. The resulting supramolecular network could modify local polarity, may weaken plant–polyphenol interactions, and may improve solvation of polyphenols, particularly those rich in hydroxyl groups [30,31]. In addition, Lane et al. [32] showed by molecular dynamics that HBA variation modifies the static and dynamic hydrogen-bonding structure, molecular organization, and heterogeneity of DES systems. Therefore, replacing or contrasting a chloride-centered ionic network with a malic acid-based formulation may introduce a different interaction landscape, as reported for organic-acid-based DES/NADES systems, which have stronger acidity, multiple carboxyl/hydroxyl sites, and markedly different thermophysical behavior, including viscosity and hydrogen-bond density [33,34]. These features are relevant because polyphenol extraction depends on a balance between solvent–solute affinity and mass-transfer constraints. A more acidic and strongly associated medium may improve the extraction or stability of phenolic acids and glycosylated flavonoids, whereas a chloride-centered ionic network may favor polyhydroxylated flavonoids or higher apparent TPC depending on matrix accessibility and water content. Under this framework, the significant effect of factor A (HBA) on all response variables, together with the universal significance of the AB interaction, is chemically coherent: the extraction behavior may not be attributed only to plant matrix or extraction time, but also to the reorganization of the eutectic supramolecular network and its interaction with the physical state of the dried leaf matrix. This framework may helps explain why the highest TPC in the present study was obtained with the ChCl-based system under freeze-drying and short extraction time, whereas the highest catechin concentration was observed in freeze-dried samples extracted for 30 min, especially in Exp. 5 and Exp. 6. Catechin is a flavan-3-ol with several phenolic hydroxyl groups, and its extraction depends not only on solvent polarity, but also on preservation against oxidation and on the accessibility of vacuolar and cell-wall-associated pools. Freeze-drying generally better preserves polyphenol and antioxidant capacity in leafy matrices by avoiding thermal degradation and excessive enzymatic or oxidative loss during dehydration [21]. This likely contributed to the strong catechin enrichment observed here. In parallel, the significance of drying type and the AB interaction suggests that catechin release was favored when the preserved microstructure of freeze-dried tissue interacted with a solvent environment capable of efficiently solvating polyhydroxylated flavonoids. The strong enrichment of quercetin + luteolin under selected freeze-dried NADES conditions is consistent with mechanistic work showing that hydrogen bonding has been proposed as a major force involved in the extraction of flavonoids from plant matrices, while solvent viscosity, polarity, and steric accessibility determine whether these interactions are effectively translated into extraction yield [35].

By contrast, chlorogenic acid, rutin, and, to some extent, neohesperidin were favored under oven-dried conditions, particularly when the malic acid-based formulation was used. A similar pattern has been reported in leaf matrices subjected to moderate thermal drying. For example, in wild guava leaves, drying at 50–60 °C resulted in the highest retention of total phenolics and selected individual compounds, with TPC reaching 145.38 mg GAE/g DL, rutin 3.20 mg/g DL at 50 °C, and chlorogenic acid 6.80 mg/g DL at 60 °C, whereas lower or higher temperatures led to greater degradation of bioactive compounds [32,36]. The authors further suggested that moderate hot-air drying may combine shorter exposure times with sufficient structural disruption to facilitate the release of polyphenols bound or compartmentalized within the tissue. Likewise, in tomato matrices, ultrasound-assisted hot-air drying retained higher levels of chlorogenic acid (0.58 mg/g) y de rutin (0.50 mg/g) than freeze-drying, supporting the idea that moderate thermal processing can reduce diffusional barriers and enhance the recovery of specific polyphenols without necessarily causing severe degradation [33,37].

Such a response is reasonable for chlorogenic acid, a phenolic acid ester, and for glycosylated flavonoids such as rutin and neohesperidin, whose extraction can benefit from greater cell-wall permeabilization and acidic media. In *Moringa oleifera* leaves, organic-acid-based DESs also showed superior polyphenols recovery compared with ethanol, and the authors attributed this to enhanced compatibility between DES polarity/acidity and the target polyphenols, while excessively viscous or sterically hindered systems performed worse [22]. Likewise, in orange peel, choline chloride:malic acid was one of the most effective NADES for TPC and antioxidant-related stability, reinforcing the idea that malic-acid-containing systems can be advantageous for acid-compatible polyphenols and for preserving extract functionality [34,38]. The present chlorogenic acid maximum of 16.05 mg/g dry leaf in Exp. 10 and the high rutin values in Exp. 10–12 are therefore consistent with a framework in which the MAc-based solvent environment, together with oven-induced matrix relaxation, preferentially favored phenolic acids and glycosylated flavonoids over catechin-rich profiles.

The behavior of antioxidant capacity is also informative. In the present study, Ax was driven primarily by HBA × drying type interaction, rather than by extraction time. This indicates that antioxidant performance was not a direct surrogate of catechin or TPC alone. A similar lack of one-to-one correspondence between global spectrophotometric responses and chromatographic composition has been reported in habanero pepper leaves and other plant systems, where different polyphenols contribute differently to radical scavenging depending on concentration, redox potential, and synergistic interactions [7,24]. Thus, the high Ax values of the oven-dried samples in the present work may be associated with the combined presence of chlorogenic acid, rutin, neohesperidin, and other co-extracted antioxidants, rather than with the maximization of a single polyphenol family. Such an effect is plausible because antioxidant mixtures may act synergistically through mechanisms including regeneration of oxidized antioxidants by other compounds, complementary radical-scavenging pathways, and the formation of additional antioxidant-active products, as discussed by Bayram and Decker [35,39]. In DPPH-based systems, this type of interaction has also been experimentally observed by Joshi et al. [36,40], who showed that combining phenolic phytochemicals with a co-occurring antioxidant matrix could enhance radical scavenging beyond the activity of the individual components.

This interpretation is also supported by the heatmap and clustering results, which separated treatments rich in chlorogenic acid/rutin/Ax from those enriched in catechin/quercetin + luteolin.

Regarding ultrasound time, the present results suggest that ExT had a limited influence on TPC and Ax, but a selective impact on specific compounds such as quercetin + luteolin, kaempferol, diosmin + hesperidin, and neohesperidin. This is consistent with the mechanistic view of UAE as a mass-transfer enhancer driven by cavitation, microstreaming, and cell disruption, but with compound-specific kinetics of release and stability. In DES/NADES systems, intermediate sonication times are often optimal because longer processing may increase diffusion only up to the point at which localized heating, prolonged exposure to radicals, or re-association effects begin to counteract extraction [22,23]. In habanero pepper leaves extracted with conventional solvents, Herrera-Pool et al. [6] also showed that ultrasound can rapidly recover polyphenols, but that solvent properties remain decisive for the final composition. Therefore, the weak time effect on TPC but significant time effects on selected molecules in the current study likely reflect the coexistence of fast-extracting compounds and compounds that require longer solvent–matrix contact or are more sensitive to the evolving physicochemical microenvironment during sonication.

The interaction analysis further reinforces this interpretation. The universal significance of AB indicates that the performance of each solvent system depended on whether the tissue had been freeze-dried or oven-dried. The selective significance of AC, BC, and ABC for kaempferol, diosmin + hesperidin, and neohesperidin suggests that these compounds were especially sensitive to the combined effects of solvent network, tissue state, and sonication process. This agrees with recent mechanistic work indicating that the extracting efficiency of NADES is governed not only by nominal polarity, but also by the number and spatial distribution of hydrogen-bonding sites, the extent of self-association within the solvent, and the dynamic availability of those sites for solute interaction [28,31]. In practical terms, the present data show that no single “best” treatment can be assumed for all responses: the best TPC, Ax, catechin, and chlorogenic acid were obtained under different conditions; therefore, individual-polyphenol-oriented process design is more informative than relying on TPC alone.

From a translational standpoint, catechin and chlorogenic acid deserve special attention because they were the two most abundant target compounds under the best-performing conditions for individual recovery. Catechins have been associated with antioxidant, anti-inflammatory, cardiometabolic, and microbiota-related benefits, and recent reviews emphasize that their clinical and nutritional relevance depends strongly on dose, matrix, and formulation [37,41]. Chlorogenic acid has likewise been linked to antioxidative, anti-inflammatory, neuroprotective, and metabolic regulatory mechanisms involving pathways such as AMPK, NF-κB, and Nrf2 [38,42]. In the present study, the best catechin-rich treatment yielded about 51.14 mg catechin/g dry leaf, whereas the best chlorogenic-acid-rich treatment yielded about 16.05 mg/g dry leaf. To provide perspective, commercial green tea extract capsules commonly provide about 160 mg catechins per daily serving (1 capsule) when standardized to 40% catechins, and some green coffee supplements provide about 200 mg chlorogenic acid per daily serving (1 capsule) when standardized to 50% chlorogenic acid [39,40,43,44]. On that basis, 1 g of dry habanero leaf under the best catechin condition would contain roughly one-third of the catechin content of such a capsule, whereas 1 g of dry leaf under the best chlorogenic-acid condition would provide roughly 8% of the chlorogenic acid declared for a commercial green coffee capsule. These are not dose-equivalence claims, because the present values correspond to extractable content in a plant matrix rather than purified standardized supplements; however, they illustrate that habanero pepper leaves can be engineered toward commercially relevant polyphenol concentrations. This is important for future applications in functional foods, nutraceuticals, cosmetic antioxidants, or polyphenol -enriched ingredients where the goal is not merely to maximize total polyphenols, but to design extracts enriched in compounds with specific technological or health-related value.

Overall, the present findings support a discussion framework in which selective extraction from *Capsicum chinense* leaves is governed by the convergence of three elements: (i) the supramolecular properties of the NADES, especially the different hydrogen-bonding environments created by choline chloride- and malic-acid-based systems; (ii) the structural changes imposed by drying; and (iii) the kinetics of release and stability under ultrasound. Under this view, habanero pepper leaves emerge as a versatile source of tailor-made polyphenol extracts, and the modulation observed here provides a basis for choosing extraction conditions according to the intended end use, whether the goal is a catechin-rich extract, a chlorogenic acid/rutin-rich extract, or an antioxidant system with broader polyphenol diversity.

## 4. Materials and Methods

### 4.1. Chemicals and Reagents

Choline chloride, fructose, sodium carbonate, Folin–Ciocalteu reagent, DPPH, gallic acid, protocatechuic acid, catechin, chlorogenic acid, p-coumaric acid, cinnamic acid, rutin, quercetin, luteolin, kaempferol, vanillin, hesperidin, neohesperidin, naringenin, apigenin, and diosmetin were purchased from Sigma-Aldrich (St. Louis, MO, USA). Methanol, acetonitrile, and acetic acid of HPLC grade were obtained from Merck/Supelco (Darmstadt, Germany) through Sigma-Aldrich (Darmstadt, Germany).

### 4.2. Raw Material

For this study, leaves were collected from greenhouse-grown habanero pepper plants of the Jaguar variety (*Capsicum chinense* Jacq.). The crop was established in the Chablekal community, Yucatán, Mexico (21°06′02.3″ N, 89°33′40.5″ W), using the regional black soil classified in the Mayan system as *Boox Lu’um*. Pruning was carried out 120 days after transplanting, overlapping with the first harvest of the fruits.

### 4.3. Drying Method of Habanero Pepper Leaves

#### 4.3.1. Freeze Drying

Leaves were freeze-dried using a Freeze dryer (Labconco Corporation, Kansas City, MO, USA). (−52 °C, 0.280 mBar, 72 h). The dried material was then ground using a Braun® coffee grinder (De’Longhi Appliances S.r.l., Treviso, Italy, model KSM-2). The resulting powder was passed through a 500 µm sieve (#35, Fisher Scientific, Boston, MA, USA) to obtain particles of uniform size, with a final moisture under 5% [45].

#### 4.3.2. Oven Drying

Leaves were placed in a Stainless-steel tray dryer (Novatech, HS60-AID model, Tlaquepaque, Jalisco, Mexico)at 44 °C for 48 h, until a moisture content below 5% was reached [5]. The grinding process was carried out as described in Section 4.3.1.

### 4.4. Evaluation of a Hydrogen Bond Acceptor and Extraction Time on Polyphenol Extraction from Habanero Pepper Leaves

#### 4.4.1. Experimental Design

To evaluate the effects of hydrogen bond acceptor (HBA) type in NADES formulation, sonication time during extraction, and leaves drying method on the response variables total polyphenol content (TPC) and antioxidant capacity (Ax), a 2 × 3 × 2 factorial design was applied. Three factors were considered. The first factor was the HBA used in the formulation of the natural deep eutectic solvent (NADES), with two levels: choline chloride (ChCl), coded as −1, and malic acid (MA), coded as + 1. The second factor was sonication time, with three levels: 10 min (−1), 20 min (0), and 30 min (+1). The third factor was drying method (type), with two levels: freeze-drying (−1) and oven-drying (+1). The design comprised a total of 12 experimental combinations covering all possible interactions among the evaluated factors, thereby allowing the assessment of the main effects, as well as the two-way and three-way interactions of HBA type, sonication time, and drying method on the response variables, as detailed in Table 2.

#### 4.4.2. Formulation of NADES Using Different Hydrogen Bond Acceptors

The methodology reported by Mansinhos et al. [46] was followed with minor modifications, and two NADES formulations were prepared. In both systems, fructose (Fru) was used as the hydrogen bond donor (HBD), while the hydrogen bond acceptor (HBA) was varied. In the first formulation, choline chloride (ChCl) was used as the HBA at a 1:1 molar ratio (ChCl:Fru). In the second formulation, malic acid (MA) was used as the HBA, also at a 1:1 molar ratio (MA:Fru). In both cases, the components were heated under constant stirring until a homogeneous phase was obtained. After cooling to room temperature, distilled water was added to achieve a 50:50 *w*/*w* ratio (NADES:water), and the mixtures were stirred until complete homogenization. The resulting NADES formulations were stored under refrigeration until use.

#### 4.4.3. Polyphenol Extraction from Habanero Pepper Leaves Using NADES with Different Hydrogen Bond Acceptors

The methodology described by Avilés-Betanzos et al. [7] was followed with some modifications. First, 1 g of leaf powder (oven-dried or freeze-dried) was weighed, and 12 mL of NADES (ChCl:Fru or MAc:Fru) was added. The mixture was then subjected to ultrasound-assisted extraction in an ultrasonic bath (Branson Ultrasonics Corporation, Danbury, CT, USA; model 351/3510, 42 kHz, 135 W), for three extraction times (10, 20, and 30 min) according to the experimental design.

Subsequently, the samples were centrifuged at 4700 rpm for 30 min at 4 °C in a centrifuge (Andreas Hettich GmbH & Co. KG, Tuttlingen, Germany; model Mikro 22-R). The recovered supernatant was then subjected to a second centrifugation in a benchtop centrifuge (Thermo Electron LED GmbH, Osterode am Harz, Germany; Heraeus Megafuge 40R model). at 15,000 rpm for 30 min at 4 °C. The newly recovered supernatants were subjected to a final centrifugation under the same conditions as in the previous centrifugation step. After the last centrifugation, the recovered supernatants were filtered through a nylon filter (0.20 µm) and transferred to amber vials.

Finally, a control extract was prepared using methanol by weighing 0.5 g of freeze-dried flour and adding 2.5 mL of 80% methanol (*v*/*v*). The same extraction times (10, 20, and 30 min) were used. The samples were then centrifuged (4700 rpm, 30 min, 4 °C), and the supernatants were recovered and filtered. Both the NADES and methanolic extracts were stored in amber vials under refrigeration until analysis.

### 4.5. Spectrophotometric Evaluation of Polyphenol Content and Antioxidant Capacity

#### 4.5.1. Determination of Total Polyphenol Content

The methodology described by Singleton et al. [47] was followed with some modifications. A 1:50 (*v*/*v*) dilution of the extracts was prepared. For each sample, 3 mL of distilled water and 250 µL of Folin reagent (1:2, *v*/*v*) were added, followed by vortex mixing, and the samples were allowed to stand for 5 min. Subsequently, 750 µL of 20% sodium carbonate (Na_2_CO_3_) and 950 µL of distilled water were added. The samples were incubated for 30 min; during the last 10 min, they were centrifuged at 4700 rpm for 10 min at 4 °C in a refrigerated centrifuge (Hettich^®^, model Mikro 22-R). Absorbance was measured at 765 nm using an UV–Vis spectrophotometer (Cole-Parmer Ltd., Stone, Staffordshire, United Kingdom; Jenway® model 6715). The results were expressed as milligrams of gallic acid equivalents per gram of dry mass (mg GAE/g DL). Prior to sample analysis, a gallic acid calibration curve was prepared over a range of 5 to 100 µg/mL (R^2^ = 0.9997; µg gallic acid/mL = 62.26x − 2.67).

#### 4.5.2. Determination of Antioxidant Capacity

The antioxidant capacity of the extracts was evaluated using the DPPH method described by Brand-Williams et al. [48], with some modifications. Briefly, 3.3 mg of DPPH reagent was weighed and diluted with methanol to a final volume of 100 mL. The solution was stirred for 10 min. After this period, the solution was measured in a UV–Vis spectrophotometer (JENWAY^®^, model 6715) at 515 nm, and its absorbance was adjusted to 0.700 ± 0.002 with methanol, as required. Once the absorbance had been adjusted, 3.9 mL of the DPPH solution was mixed with 100 µL of each sample. The mixtures were then allowed to stand for 30 min. During the last 10 min, the samples were centrifuged at 4700 rpm and 4 °C (Hettich^®^, model Mikro 22-R). Finally, the absorbance of each sample was measured at 515 nm using the same UV–Vis spectrophotometer (JENWAY^®^, model 6715). Results were expressed as the percentage of DPPH radical inhibition using Equation (1):

(1) $$\%\;\mathrm{Inhibition}\;=\;\left(\frac{A_{0}-A_{s}}{A_{0}}\right)\times 100$$

where

A_0_ = absorbance of the DPPH Adjusted solution (without sample)

A_s_ = absorbance of the sample (DPPH + sample)

### 4.6. Chromatographic Analysis of NADES-Based Extracts

Individual polyphenols in habanero pepper leaf extracts were quantified following the procedure described by Chel-Guerrero et al. [5]. Analyses were carried out using an Acquity UPLC H-Class system (Waters Corporation, Milford, MA, USA) fitted with a diode array detector (DAD), an Acquity UPLC HSS C18 column (Waters Corporation, Wexford, Leinster, Ireland), and Empower 3 chromatography data software (Waters Corporation, Milford, MA, USA). A stock solution of each standard (1 mg/mL) was used to construct calibration curves within a concentration range of 1–75 μg/mL. The standard mixture comprised 15 polyphenolic compounds (Sigma-Aldrich, St. Louis, MO, USA), including the phenolic acids gallic acid, protocatechuic acid, chlorogenic acid, coumaric acid, cinnamic acid, as well as the flavonoids catechin, rutin, naringenin, diosmetin, apigenin, kaempferol, quercetin + luteolin and diosmin + hesperidin, with the latter two pairs quantified together because of chromatographic co-elution.

Chromatographic separation was performed at 45 °C using an injection volume of 2 μL, and detection was monitored at 280 nm. The mobile phases consisted of water containing 0.2% acetic acid (A) and acetonitrile containing 0.1% acetic acid (B). The gradient program started at 1% B (99% A) and increased gradually to 30% B (70% A) over the first 10 min. This composition was held constant from 10 to 12 min, after which the system was returned to the initial conditions over the final 3 min.

Only those individual polyphenols detected in each extract and identified by comparison with the retention times of the 15 reference standards were quantified.

### 4.7. Statistical Analysis

All experimental treatments were performed in triplicate in a randomized order. Results were expressed as mean ± standard deviation. Data were analyzed using a multifactorial ANOVA according to the factorial structure of the experimental design. The model included the main effects of hydrogen-bond acceptor (HBA), drying method (D), and extraction time (T), as well as their two-way and three-way interactions:

$$Y_{ijkm}=\mu +HBA_{i}+D_{j}+T_{k}+(HBA\times D{)}_{ij}+(HBA\times T{)}_{ik}+(D\times T{)}_{jk}+(HBA\times D\times T{)}_{ijk}+\varepsilon_{ijkm}$$

where $Y_{ijkm}$ represents each response variable, μ is the overall mean, $HBA_{i}$ is the effect of the hydrogen-bond acceptor, $D_{j}$ is the effect of the drying method, $T_{k}$ is the effect of extraction time, and $\varepsilon_{ijkm}$ is the residual error.

When significant effects were detected, mean comparisons were performed using the least significant difference (LSD) test at a 95% confidence level (*p* < 0.05). The LSD test was used as a protected post hoc procedure, applied only after significant ANOVA effects, to identify specific differences among predefined treatment combinations within the factorial design.

To estimate the relative importance of each factor and interaction, the percentage contribution was calculated from the ANOVA sums of squares using Equation (2):

(2) $$\%\;\mathrm{contribution}=\frac{SS_{\mathrm{factor}\;\mathrm{or}\;\mathrm{interaction}}}{SS_{\mathrm{total}\;\mathrm{corrected}}}\times 100$$

where $SS_{\mathrm{factor}\;\mathrm{or}\;\mathrm{interaction}}$ is the sum of squares of each main factor or interaction, and $SS_{\mathrm{total}\;\mathrm{corrected}}$ is the corrected total sum of squares. Results are presented in Table A2.

Before interpreting the ANOVA results, model assumptions were evaluated. Normality of residuals was assessed using the Shapiro–Wilk and Kolmogorov–Smirnov tests, while homogeneity of variance was evaluated using Levene’s test. When deviations from normality were detected, the ANOVA results were interpreted with caution, particularly for individual chromatographic polyphenols, considering the balanced factorial design and the absence of severe variance heterogeneity.

Hierarchical clustering and heatmap analysis were performed using z-score standardized data to explore the relationship among treatments and response variables, including total phenolic content, antioxidant capacity, and individual polyphenols. Pareto chart analysis was used to visualize the standardized effects of the experimental factors and their interactions. Statistical analyses, Pareto charts, clustering, heatmap generation, and overall data processing were performed using Statgraphics Centurion XVII.II-X64 software (Statgraphics Technologies Inc., The Plains, VA, USA) and Google Colab (Google LLC, Mountain View, CA, USA).

## 5. Conclusions

The present study demonstrates that UAE/NADES extraction can be used as a tunable platform to direct the selective recovery of polyphenols from *Capsicum chinense* leaves. The results indicate that the hydrogen bond acceptor, drying method, and extraction time influenced the compound-level phenolic profile, suggesting that NADES formulation and processing conditions can be adjusted to favor the recovery of specific phenolic compounds. This is relevant because extraction performance should not be evaluated only through global responses such as total polyphenol content or antioxidant capacity, since individual polyphenols respond differently to the extraction system. In practical terms, these results indicate that habanero pepper leaves, an underutilized agro-industrial by-product, can be valorized as a source of polyphenol-enriched extracts with tailored compositions. Thus, the selection of extraction conditions should be guided by the intended application, whether the objective is to obtain extracts enriched in specific flavonoids, phenolic acids, or broader antioxidant profiles for future use in food, nutraceutical, cosmetic, or pharmaceutical formulations.

## Figures and Tables

**Table 1:** Significance of main factors and interactions on TPC, Ax, and polyphenolic composition.

Response Variables									
	TPC	Ax	Ctn	ChAc	Rt	Q + L	Kmp	D + H	NeHe
**Main Factors**									
**A**	<0.0001	0.0008	<0.0001	<0.0001	<0.0001	<0.0001	0.0128	0.0002	<0.0001
**B**	0.0630	<0.0001	<0.0001	<0.0001	<0.0001	<0.0001	0.0116	0.0304	<0.0001
**C**	0.9463	0.0642	0.1310	0.1090	0.1090	0.0060	0.0033	0.0019	0.0020
**Interactions**									
**AB**	<0.0001	<0.0001	<0.0001	<0.0001	0.0131	0.0131	<0.0001	0.0304	<0.0001
**AC**	0.1327	0.2833	0.0702	0.3273	0.1231	0.1231	0.0030	0.0019	0.0017
**BC**	0.0004	0.1000	0.0658	0.9686	0.0578	0.0578	0.0035	0.0047	0.0018
**ABC**	0.0001	0.1842	0.1024	0.2856	0.0143	0.0143	0.0032	0.0047	0.0018

Note: A = Hydrogen Bond Acceptor; B = Drying type; C = Extraction time; TPC = Total polyphenol content; Ax = Antioxidant capacity; Ctn = Catechin; ChAc = Chlorogenic acid; Rt = Rutin; Q + L = Quercetin + luteolin; Kmp = Kaempferol; D + H = Diosmin + hesperidin; NeHe = Neohesperidin. Multifactorial ANOVA was performed at a 95% confidence level (*p* < 0.05).

**Table 2:** Factorial design 2 × 3 × 2 for evaluating the effect of hydrogen bond acceptor, extraction time, and drying method on the total polyphenol content and antioxidant capacity of habanero pepper leaves.

Exp	Encoded Values			Real Values			Response Variables	
	X_1_	X_2_	X_3_	HBA	ExT	DMe	TPC	Ax
**1**	−1	−1	−1	ChCl	10	Freeze Drying	36.18 ± 0.70 ^h^	84.62 ± 0.44 ^bc^
**2**	−1	0	−1	ChCl	20	Freeze Drying	34.57 ± 0.94 ^g^	84.45 ± 1.22 ^bc^
**3**	−1	1	−1	ChCl	30	Freeze Drying	32.76 ± 0.93 ^f^	84.60 ± 1.49 ^bc^
**4**	1	−1	−1	MAc	10	Freeze Drying	26.31 ± 0.58 ^b^	80.71 ± 0.52 ^a^
**5**	1	0	−1	MAc	20	Freeze Drying	26.72 ± 0.45 ^b^	81.00 ± 0.71 ^a^
**6**	1	1	−1	MAc	30	Freeze Drying	27.02 ± 0.12 ^b^	80.95 ± 2.26 ^a^
**7**	−1	−1	1	ChCl	10	Oven Drying	30.29 ± 1.28 ^de^	83.48 ± 0.88 ^b^
**8**	−1	0	1	ChCl	20	Oven Drying	30.88 ± 0.65 ^e^	87.24 ± 0.55 ^de^
**9**	−1	1	1	ChCl	30	Oven Drying	32.68 ± 1.11 ^f^	86.43 ± 0.65 ^de^
**10**	1	−1	1	MAc	10	Oven Drying	28.68 ± 1.41 ^c^	86.38 ± 0.66 ^de^
**11**	1	0	1	MAc	20	Oven Drying	28.68 ± 0.59 ^c^	85.90 ± 0.91 ^cd^
**12**	1	1	1	MAc	30	Oven Drying	29.08 ± 1.02 ^cd^	86.95 ± 0.18 ^de^

Note: Exp = experiment; HBA = hydrogen bond acceptor; ChCl = Cholin chloride; MAc = Malic acid; ExT = Extraction time (min); DMe = Drying method; TPC = total polyphenol content; Ax = antioxidant capacity. Different letters in the same column indicate statistical differences (LSD, *p* < 0.05).

## Data Availability

The original contributions presented in the study are included in the article. Further inquiries can be directed to the corresponding authors.

## Appendix A

**Table A1:** Polyphenol profile of extracts obtained under the 2 × 3 × 2 experimental design.

Experiment	Encoded Values			Polyphenol Profile mg/g Dry Leaf						
	X_1_	X_1_	X_1_	Catequina	Ac. Clorogénico	Rutina	D + H	Neohesperidina	Q + L	Kaempferol
1	−1	−1	−1	0.84 ± 0.04 ^e^	ND	0.36 ± 0.07 ^fg^	ND	ND	2.61 ± 0.01 ^fg^	2.45 ± 0.03 ^b^
2	−1	0	−1	0.91 ± 0.04 ^e^	ND	0.24 ± 0.04 ^fg^	ND	ND	2.61 ± 0.00 ^g^	2.47 ± 0.02 ^ab^
3	−1	1	−1	0.65 ± 0.05 ^e^	ND	0.20 ± 0.00 ^g^	ND	ND	2.61 ± 0.02 ^g^	2.47 ± 0.02 ^ab^
4	1	−1	−1	34.89 ± 0.28 ^b^	2.57 ± 0.03 ^d^	1.07 ± 0.03 ^e^	0.14 ± 0.00 ^de^	5.06 ± 0.02 ^bc^	5.37 ± 0.03 ^a^	2.51 ± 0.00 ^a^
5	1	0	−1	51.14 ± 0.76 ^a^	2.99 ± 0.14 ^d^	1.14 ± 0.01 ^de^	0.19 ± 0.01 ^d^	4.99 ± 0.12 ^bcd^	5.36 ± 0.01 ^a^	ND
6	1	1	−1	47.88 ± 1.23 ^a^	2.67 ± 0.02 ^d^	1.25 ± 0.01 ^d^	0.21 ± 0.02 ^d^	5.04 ± 0.01 ^bcd^	4.75 ± 0.02 ^b^	ND
7	−1	−1	1	1.06 ± 0.03 ^e^	ND	1.60 ± 0.13 ^c^	ND	ND	2.65 ± 0.03 ^fg^	ND
8	−1	0	1	ND	1.95 ± 0.31 ^e^	ND	ND	5.16 ± 0.21 ^ab^	3.09 ± 0.51 ^e^	ND
9	−1	1	1	ND	1.13 ± 0.03 ^f^	ND	ND	5.37 ± 0.05 ^a^	ND	ND
10	1	−1	1	32.08 ± 3.50 ^b^	16.05 ± 0.09 ^a^	5.86 ± 0.09 ^a^	0.11 ± 0.05 ^e^	4.92 ± 0.01 ^cd^	3.26 ± 0.04 ^de^	2.47 ± 0.01 ^b^
11	1	0	1	18.13 ± 2.07 ^d^	12.18 ± 0.44 ^c^	4.57 ± 0.02 ^b^	0.12 ± 0.00 ^e^	4.82 ± 0.01 ^d^	3.31 ± 0.08 ^de^	2.45 ± 0.01 ^b^
12	1	1	1	22.28 ± 0.51 ^c^	14.94 ± 0.05 ^b^	5.92 ± 0.05 ^a^	1.36 ± 0.07 ^a^	4.89 ± 0.00 ^cd^	3.03 ± 0.14 ^ef^	2.46 ± 0.00 ^b^
MeOH10	Control			1.36 ± 0.02 ^e^	ND	0.38 ± 0.04 ^f^	0.41 ± 0.02 ^b^	2.11 ± 0.11 ^e^	3.65 ± 0.05 ^cd^	0.53 ± 0.01 ^c^
MeOH20	Control			1.28 ± 0.05 ^e^	ND	0.32 ± 0.0 ^fg^	0.31 ± 0.00 ^c^	2.10 ± 0.10 ^e^	3.57 ± 0.01 ^cd^	0.57 ± 0.02 ^c^
MeOH30	Control			1.41 ± 0.00 ^e^	ND	0.32 ± 0.01 ^fg^	0.36 ± 0.03 ^bc^	1.99 ± 0.01 ^e^	3.75 ± 0.05 ^c^	0.55 ± 0.01 ^c^

**Note:** X_1_ = Hydrogen bond acceptor; X_2_ = Extraction time; X_3_ = Drying method; ND = Not detected. Values are means ± standard deviation (n = 3). Different letters in the same column indicate statistical differences (LSD, *p* < 0.05).

**Table A2:** Percentage contribution of the main experimental factors and interactions to TPC, Ax, and individual polyphenols.

Response Variable	Main Contributing Effects
**TPC**	HBA = 67.61%; HBA × drying method = 18.31%; residual = 5.38%
**Ax**	Drying method = 46.38%; HBA × drying method = 19.80%; residual = 12.93%; HBA = 9.16%
**Catechin**	HBA = 59.96%; drying method = 18.22%; HBA × drying method = 17.20%
**Chlorogenic acid**	HBA = 47.48%; drying method = 29.37%; HBA × drying method = 20.64%
**Rutin**	HBA = 45.13%; drying method = 27.91%; HBA × drying method = 21.73%
**Quercetin + luteolin**	HBA = 46.61%; drying method = 22.29%; extraction time = 9.96%; HBA × drying method × extraction time = 7.39%
**Kaempferol**	HBA × drying method = 70.42%; HBA × extraction time = 6.03%; extraction time = 5.91%
**Diosmin + hesperidin**	HBA = 23.42%; extraction time = 17.37%; HBA × extraction time = 17.37%; drying method × extraction time = 15.02%; HBA × drying method × extraction time = 15.02%
**Neohesperidin**	HBA = 45.29%; HBA × drying method = 14.89%; drying method = 12.43%

**Note:** TPC = total phenolic content; Ax = antioxidant capacity; HBA = hydrogen-bond acceptor. Only the main contributing effects are shown for each response variable. Individual phenolic compounds were quantified by UPLC-DAD and reported as mg/g dry leaf.
